# Development of a Taekwondo Combat Model Based on Markov Analysis

**DOI:** 10.3389/fpsyg.2019.02188

**Published:** 2019-10-01

**Authors:** Cristina Menescardi, Coral Falco, Concepción Ros, Verónica Morales-Sánchez, Antonio Hernández-Mendo

**Affiliations:** ^1^AFIPS Research Group, Department of Teaching of Musical, Visual and Corporal Expression, University of Valencia, Valencia, Spain; ^2^Department of Sport, Food and Natural Sciences, Western Norway University of Applied Sciences, Bergen, Norway; ^3^Grupo de Investigación en Educación para una Actividad Física Saludable, Departamento de Gestión y Didáctica de la Actividad Física, Facultad de Ciencias de la Actividad Física y el Deporte, Universidad Católica de Valencia “San Vicente Mártir”, Valencia, Spain; ^4^Department of Social Psychology, Social Work, Anthropology and East Asian Studies, University of Málaga, Málaga, Spain

**Keywords:** tactical patterns, probabilistic models, martial arts, statistics, combat sports

## Abstract

The purpose of the present study was to examine male and female Olympic taekwondo competitors’ movement patterns according to their tactical actions by applying a Markov processes analysis. To perform this study, 11,474 actions by male competitors and 12,980 actions by female competitors were compiled and analyzed. The results yielded 32 significant sequences among male competitors and 30 among female competitors. Male competitors demonstrated 11 sequences initiated by an attack, 11 initiated by a counterattack, and 10 initiated by a defensive action. Female competitors demonstrated nine sequences initiated by an attack, 11 initiated by a counterattack, and 10 initiated by a defensive move. The five most popular sequences were the opening and dodge, the direct attack and simultaneous counterattack, the dodge with a direct attack, the indirect attack and simultaneous counterattack, and the simultaneous counterattack with a direct attack. Markov chains help provide coaches and researchers with relevant information about the frequency of actions, both in terms of their frequency of occurrence and the order of their occurrence, during a real competition. It is suggested that coaches and athletes focus on these patterns when training for a real competition.

## Introduction

In elite sports, performance needs to be understood from different perspectives, including a tactical perspective. Tactical decisions made during combat are not directly observable during competition; only the tactical actions performed by athletes can provide an indication of the tactics that have been applied. To make tactical decisions during competition, competitors must have knowledge of the different tactical options and their probability of success, so they can select the correct action to perform and the proper moment to act (O’Donoghue, 2010). Normally, a tactical scheme is prepared to face combat situations in order to take the opponents by surprise by using the specific tactical actions (Gréhaigne and Godbout, 1995). Tactics provide the context that justifies techniques (Gréhaigne et al., 2001, 2005), because tactical actions involve not only motor skills to ensure efficient execution but also the choice to perform the appropriate action (Gréhaigne et al., 2001) in each combat situation. Tactical actions in taekwondo are usually subdivided into offensive (i.e., attacks and counterattacks) and defensive actions according to the purpose (i.e., score or avoid being scored against) of the athlete (Menescardi et al., 2019). The tactics applied during competitions have to be found in objective data (O’Donoghue, 2010).

Objective data could be based on stochastic models (i.e., a mathematical representation of a system which specifies the probable occurrence of a series, or set, of event states) that prove to be profitable analytic tools in the study of sports because they lend themselves to the prediction of future events from past data. Specifically, Markov chains are stochastic models that describe a sequence of possible events in which the probability of each event depends only on the state attained in the previous event (McGarry and Franks, 1994; Castellano et al., 2007). In short, a stochastic process exhibits Markov properties if the present state contains sufficient historical information from which predict the next event (Gottman and Roy, 1990).

In this regard, Markov chains help coaches and researchers to understand the way athletes behave in competition, as well as the relationships between actions (Ozcinar and Ozturk, 2013). They show whether one behavior increases or decreases the probability that another behavior will occur (Yoder and Tapp, 2004), which may not be explained by chance (Ozcinar and Ozturk, 2013). Markov chain analyses have been applied in the field of sports to describe and predict playing profiles or patterns (McGarry and Franks, 1994; Barnett and Pollard, 2011; Goldner, 2012) to determine the relevance to performance of tactical patterns (Barnett and Pollard, 2011). In adversary sports, McGarry and Franks (1994) used Markov chains to study the interactions between players in squash and to predict match behaviors and outcome probabilities.

In combat sports, this method of analysis has proven to be a novel approach as part of an increased interest in studying the relationships between actions performed by competitors (González-Prado et al., 2015; Miarka et al., 2015; Menescardi and Estevan, 2017; Tarragó et al., 2017). In fencing, it has been shown that a pattern of pressing action was followed by the preparation and attacking action, while counterattacking actions were performed by the opponent (Tarragó et al., 2017). In judo, the observed patterns reflected four situations (i.e., attack to the front, to the right, to the rear, and to the left) such that the sequences finished in either groundwork, a moment of pause, or the end of combat (Miarka et al., 2015). Just as in fencing and judo, in taekwondo it is important to know the previous action and how to respond to it, and when to initiate a sequence intending to score.

In the taekwondo field, the finals of six male Championships and World Cups (from 2000 to 2008) were analyzed by González-Prado et al. (2015). They found a relationship between offensive and defensive actions which follow one another during the bout. Menescardi and Estevan (2017) also proved the utility of lag sequential analysis in identifying the succession of attacks and counterattacks during bouts. They found a significant relationship between attacks (direct and indirect) and a simultaneous counterattack; they also found that indirect attacks had a significant relationship with dodges. In addition, anticipatory, simultaneous, and posterior counterattacks had a significant relationship with direct and indirect attacks, and posterior counterattacks were also significantly associated with dodges (Menescardi and Estevan, 2017).

Research that analyses behavioral patterns in taekwondo by using Markov chains is rare and focused only on the sample of male competitors. Previous studies (Casolino et al., 2012; Ferreira-Santos et al., 2014) have also found different patterns in relation to sex. Therefore, the aim of the present study was to examine behavioral tactical patterns of Olympic taekwondo competitors, in a sample of both male and female competitors, based on Markov processes analysis. Specifically, Markov chain is a technique for analyzing the relationships between two immediate key behaviors (focal and conditioned), offering the information needed for the decision-making of the selection of its immediately following behavior and offering athlete’s action–response profiles (McGarry and Franks, 1994; Pfeiffer et al., 2010). This information can be used to improve athlete’s tactical domain learning of decision-making. In a combat sport, as taekwondo, were actions are dependent on the opponents (Casolino et al., 2012) the technique can help coaching decisions based on “if-then” statements (McPherson and Thomas, 1989; McPherson, 1994; Gréhaigne and Godbout, 1995; Gréhaigne et al., 2001). This highlights the relevance of this technique in taekwondo. To test the hypothesis of whether technical–tactical patterns with greater probability of occurrence than chance based on Markov chain could explain the athlete’s behavior in Olympic competition, this study describes the tactical actions that occur just before or after another tactical action (attacks, counterattacks, and defenses) by using a Markov process analysis. These analyses enable coaches and psychologists to train both offensive and defensive strategies (Maneiro Dios and Amatria Jiménez, 2018), specifically for each tactical action and their subsequent one, according to their major occurrence in competition.

## Materials and Methods

### Methodology and Design

An observational methodology was used in a natural context. The design was diachronic, nomothetic, and multidimensional (Anguera et al., 2011). The natural context allowed us to analyze the sequence, the association, and the covariation relationship between behaviors (Anguera, 2010). A diachronic design allowed us to understand the order in which actions occurred, in terms of prospective (from one action to the following one) analysis. Nomothetic design implies the behavior analysis of some participants while multidimensional design implies there are specific criteria under study (Anguera et al., 2011; Arias-Pujol and Anguera, 2017; Castañer et al., 2017).

### Participants and Sample

In total, 151 matches from the London Olympic Games in 2012, involving both male (*n* = 75) and female (*n* = 76) competitors, were studied to analyze tactical actions. One male bout was not completed because of the injury of one competitor. This research was carried out in accordance with the Declaration of Helsinki and the Belmont Report. Because the videotapes analyzed in the present study are in the public domain, it was not necessary to acquire informed consent from the athletes observed (National Institutes of Health, 1978; American Psychological Association, 2002). The study protocol was approved by the Human Research Ethics Committee of the University of Valencia.

### Materials

For codifying tactical actions in taekwondo, the taekwondo observational tool (TKDOT) validated by Menescardi et al. (2017) was used (Table 1). All coding was carried out using HOISAN 1.5.6 software (Hernández-Mendo et al., 2012). Data were codified as multi-events to perform the posterior Markov sequential analysis.

**TABLE 1:** Criteria, categories, and categorical core of the TKDOT.

**Criterion**	**Categories**	**Categorical core**
	Opening (OPE)	Movement to get control of the distance with the opponent or bridge the gap between both competitors.
	Direct attack (DIA)	Offensive action without previous movement ending with an impact on the opponent’s body.^∗∗^
	Indirect attack (INA)	Offensive action with previous movement such as a step, skip, opening, guard change, kicking trajectory modification, etc., ending with an impact on the opponent’s body.^∗∗^
	Anticipated counterattack (ACA)	Action that starts during the opponent’s attack. The athlete kicks the attacker during the preparatory phase (guard) and/or initial phase (when the opponent’s knee is being raised).^∗∗^
Tactical action	Simultaneous counterattack (SCA)	Action that starts at the same time as the opponent’s attack. The athlete kicks at the same time as the opponent. Thus, the counter attacker kicks at the end of the attacker’s initial phase (leg raised) or during the impact momentum (impact phase) of the attacker’s kick.^∗∗^
	Posterior counterattack (PCA)	Action that begins after the opponent’s attack (during the descending phase, or when attacker’s leg touches the ground). The athlete kicks at the same time as the opponent. This action (sometimes) includes a previous backward displacement to dodge the opponent’s attack.^∗∗^
	Block (BLO)	Defensive actions to avoid the impact of a kick by placing one arm or leg between the protector and the leg of the opponent.^∗^
	Dodge (DOD)	Defensive movement to avoid being kicked by the opponent.^∗^
	Cut (CUT)	Defensive forward movement to avoid being beaten by a close opponent, and to prevent the attacking action from being completed.^∗^

*^∗^This action does not have a scoring objective. ^∗∗^This action is performed with the purpose of scoring.*

### Intra- and Inter-Observer Reliability

Six observers were divided into two groups (groups A and B) to analyze the reliability of the data. To evaluate the inter-observer reliability, each observer analyzed six bouts. To evaluate the intra-observer reliability, one of the observers analyzed the same six bouts twice in a row. Cohen’s Kappa (κ) was used to calculate intra- and inter-observer reliability. Values between 0 and 0.20 show weak conformity, values between 0.21 and 0.40 show distant conformity, values between 0.41 and 0.60 show moderate conformity, values between 0.61 and 0.80 show strong conformity, and values between 0.81 and 1 show an almost perfect conformity, following previous studies (Miarka et al., 2015). The inter- and intra-observer results yielded Cohen’s kappa values >0.85, showing an almost perfect conformity.

### Tactical Modeling Through the Markov Model

**FIGURE 1:**
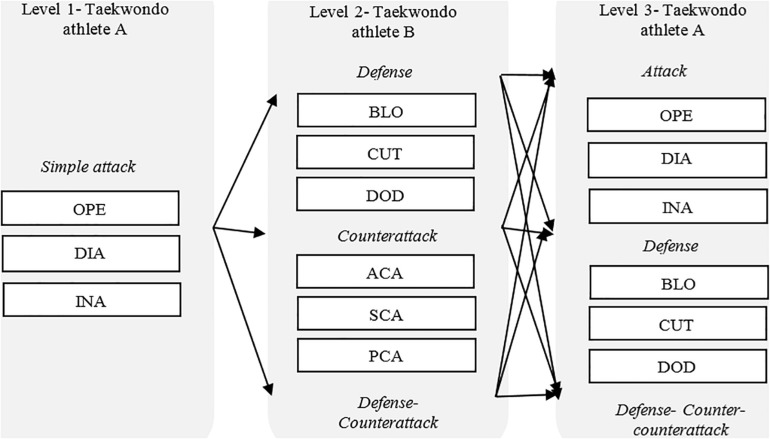
Tactical offensive–defensive sequence of three levels for taekwondo (modified from Szabó, 1977).

Markov sequential analysis of one lag was performed. Lag zero was established as the focal behavior, and the following lag (lag 1) corresponded with the first action (conditioned behavior) that followed the focal behavior. These focal behaviors were attacks, counterattacks, and defensive actions following the tactical scheme proposed (Figure 1) for taekwondo, based on the tactical scheme stated by Szabó (1977) for fencing. Every tactical action was considered as conditioned behavior.

The Markov model and transition probabilities were examined using a Pearson’s Chi-square statistic (χ^2^) and G-test (*G*^2^) to compare observed and expected transition frequencies (Haccou and Meelis, 1992). For each possible event pairing (focal and conditioned behaviors), *z*-scores were computed to reveal transitional probabilities that were significantly higher and lower than the expected probability (Bakeman and Quera, 2011; Anguera et al., 2018). A *z*-score > 1.96 at 0.05 alpha level was then used to determine significant relationships between behaviors. Statistical analysis was conducted with SDIS-GSEQ v.5.1. software. Another advantage of this software is that it allows researchers to determine the frequency of its use, solving one of the problems of Markovian models.

## Results

**FIGURE 2:**
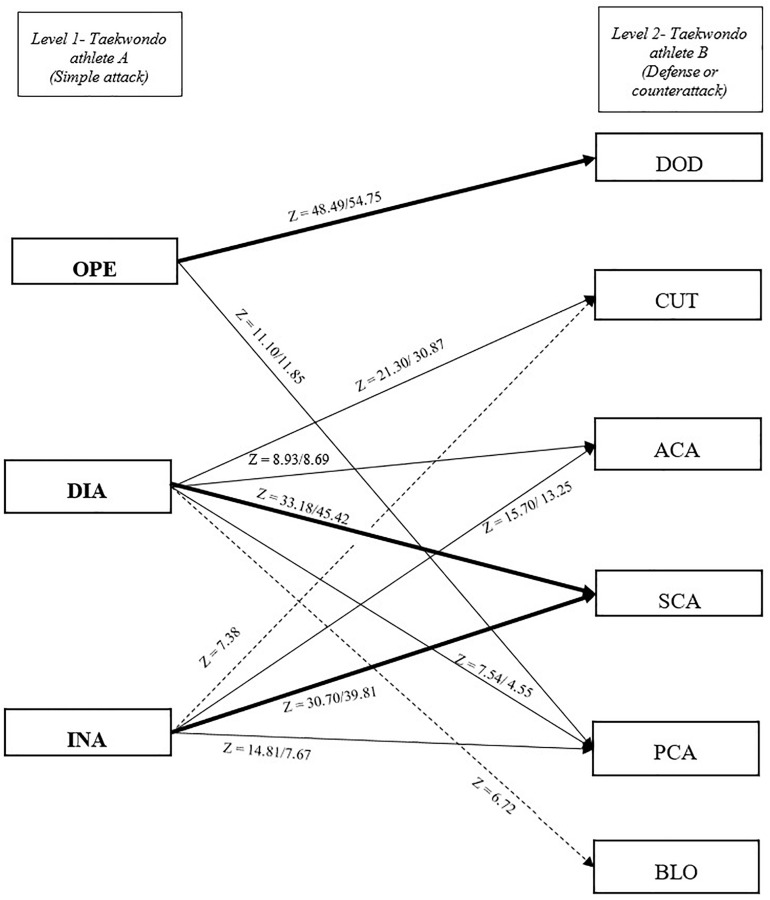
Tactical model for taekwondo competitors with *z* significant values for each pair of tactical actions, attacks, and counterattacks or defensive actions. *Z*-scores (adjusted residuals) indicate male/female values. A straight arrow indicates a significant relationship between tactical actions in both genders. A bold arrow indicates a strong relationship (*z* > 30). A discontinuous arrow indicates the relationship between tactical actions only in male competitors. OPE, opening; DOD, dodge; DIA, direct attack; INA, indirect attack; BLO, block; CUT, cut; SCA, simultaneous counterattack; ACA, anticipatory counterattack; PCA, posterior counterattack.

**FIGURE 3:**
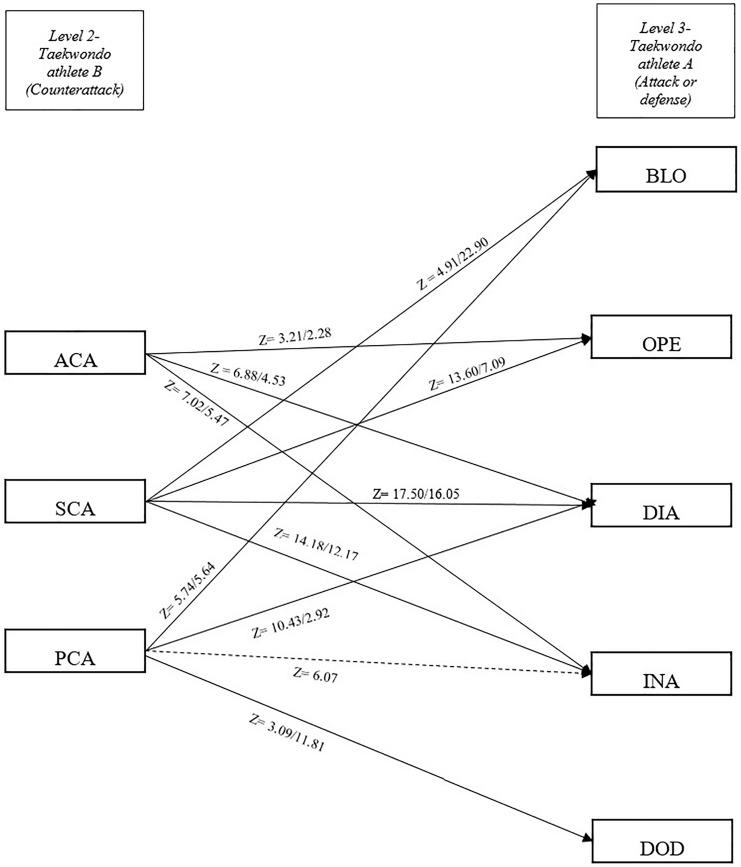
Tactical model for taekwondo competitors with *z* significant values for each pair of tactical actions, counterattacks, and attacks or defensive actions. *Z*-scores (adjusted residuals) indicate male/female values. A straight arrow indicates significant relationship between tactical actions in both genders. A discontinuous arrow indicates the relationship between tactical actions only in male competitors. OPE, opening; DOD, dodge; DIA, direct attack; INA, indirect attack; BLO, block; CUT, cut; SCA, simultaneous counterattack; ACA, anticipatory counterattack; PCA, posterior counterattack.

**FIGURE 4:**
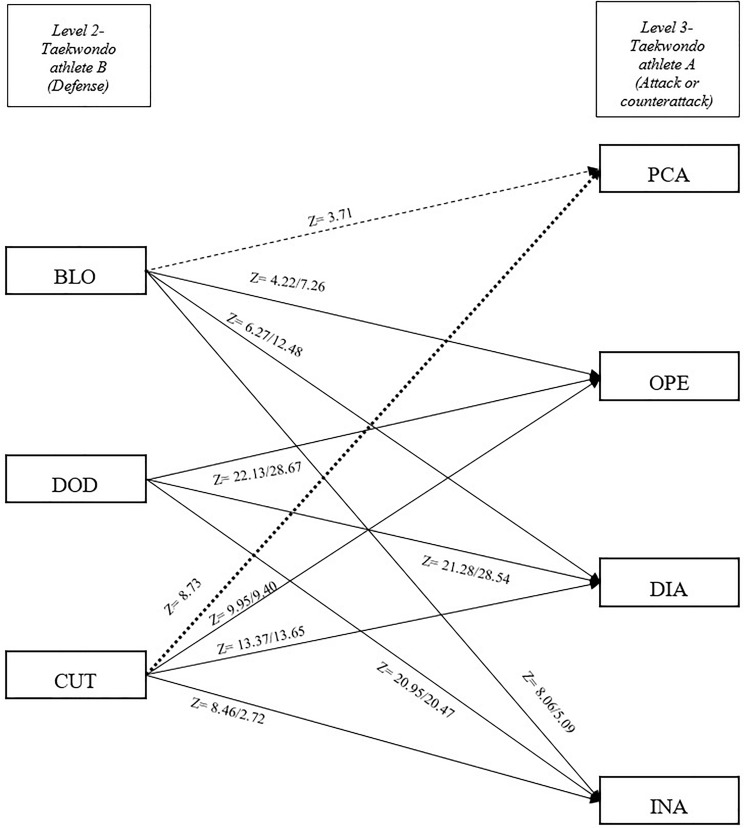
Tactical model for taekwondo competitors with *z* significant values for each pair of tactical actions, defensive actions, and attacks or counterattacks. *Z*-scores (adjusted residuals) indicate male/female values. A straight arrow indicates significant relationship between tactical actions in both genders. A discontinuous arrow indicates the relationship between tactics only in male while a dotted arrow indicates the relationship between tactical actions only in female competitors. OPE, opening; DOD, dodge; DIA, direct attack; INA, indirect attack; BLO, block; CUT, cut; SCA, simultaneous counterattack; ACA, anticipatory counterattack; PCA, posterior counterattack.

Male competitors performed a total of 11,474 actions, which were categorized into 1,460 openings, 2,281 direct attacks, 1,711 indirect attacks, 269 anticipatory counterattacks, 1,647 simultaneous counterattacks, 935 posterior counterattacks, 537 blocks, 723 cuts, and 1,911 dodges. Female competitors performed 12,980 actions, subdivided into 1,704 openings, 2,752 direct attacks, 1,316 indirect attacks, 182 anticipatory counterattacks, 2,422 simultaneous counterattacks, 649 posterior counterattacks, 834 blocks, 692 cuts, and 2,429 dodges. Z-scores are shown in Table 2. The number of occurrences for each paring event is shown in Table 3. In this table, a total of 32 patterns for male competitors are shown that explain 9,405 sequences and 30 patterns for female competitors are shown that explain 10,520 sequences (Table 3). The tactical models for male and female competitors based on Markovian processes appear in Figures 2–4 in relation to offensive (both attacks and counterattacks) and defensive actions, respectively. Regarding the level 1 of the tactical model (taekwondo athlete A), OPE was followed by PCA and DOD in both genders (level 2, actions performed by taekwondo athlete B). DIA was followed by ACA, SCA, PCA, and CUT in both genders and BLO in males. INA was followed by ACA, SCA, and PCA in both genders and CUT in males. Regarding the level 2 of the tactical model (taekwondo athlete B), ACA was followed by OPE, DIA, and INA in both genders and BLO in females (level 3, actions performed by taekwondo athlete A). SCA was followed by OPE, DIA, INA, and BLO in both genders. PCA was followed by DIA, BLO, and DOD in both genders and INA in males. BLO, DOD, and CUT were followed by OPE, DIA, and INA in both genders and BLO were also followed by PCA in males, while CUT was also followed by PCA in females.

**TABLE 2:** *Z*-scores for each possible event pairing in relation to gender.

		**OPE**	**DIA**	**INA**	**ACA**	**SCA**	**PCA**	**BLO**	**DOD**	**CUT**
		
		**Male**								
Taekwondo athlete A	OPE	−14.28	−17.86	−15.02	−2.37	−7.40	**11.10**	−7.47	**48.49**	1.36
	DIA	−16.85	−21.99	−19.21	**8.93**	**33.18**	**7.54**	**6.72**	−2.26	**21.30**
	INA	−15.58	−20.99	−17.71	**15.70**	**30.70**	**14.81**	0.05	1.28	**7.38**
Taekwondo athlete B	ACA	**3.21**	**6.88**	**7.02**	−2.55	−6.42	−4.92	0.76	−4.06	−3.52
	SCA	**13.60**	**17.50**	**14.18**	−6.70	−17.39	−12.82	**4.91**	−11.90	−9.15
	PCA	1.71	**10.43**	**6.07**	−4.66	−12.85	−9.29	**5.74**	**3.09**	−5.64
	BLO	**4.22**	**6.27**	**8.06**	−3.65	−8.15	**3.71**	−2.48	−7.09	−4.47
	DOD	**22.13**	**21.28**	**20.95**	−7.34	−18.95	−12.76	−6.20	−20.84	−10.44
	CUT	**9.95**	**13.37**	**8.46**	−4.01	−10.87	−1.72	−3.18	−11.42	−6.68
		
		χ^2^ = 10478.15		*df* = 64	*p* < 0.01	*G*^2^ = 11607.89		*df* = 64	*p* < 0.01	

		**Female**								

Taekwondo athlete A	OPE	−14.29	−21.01	−13.30	−1.87	−6.27	**11.85**	−11.27	**54.75**	−6.19
	DIA	−20.76	−28.16	−17.70	**8.69**	**45.42**	**4.55**	−1.34	−7.16	**30.87**
	INA	−14.49	−19.26	−12.24	**13.25**	**39.81**	**7.67**	−5.58	−3.77	1.58
Taekwondo athlete B	ACA	**2.28**	**4.53**	**5.47**	−1.61	−6.07	−3.11	**3.52**	−3.24	−2.55
	SCA	**7.09**	**16.05**	**12.17**	−6.26	−25.63	−12.16	**22.90**	−6.83	−8.72
	PCA	−1.18	**2.92**	1.89	−3.11	−12.51	−5.83	**5.64**	**11.81**	−3.74
	BLO	**7.26**	**12.48**	**5.09**	−3.21	−11.61	1.65	−0.80	−8.05	−4.80
	DOD	**28.67**	**28.54**	**20.47**	−5.59	−25.16	−10.88	−8.06	−25.64	−10.22
	CUT	**9.40**	**13.65**	**2.72**	−3.20	−11.75	**8.73**	−4.80	−9.76	−6.24
		
		χ^2^ = 13283.10		*df* = 64	*p* < 0.01	*G*^2^ = 14009.61		*df* = 64	*p* < 0.01	

*First column indicates focal behavior while first row indicates conditioned behavior (action that follow the focal behavior). Significant relationships (*z* > 1.96) are in bold. OPE, opening; DOD, dodge; DIA, direct attack; INA, indirect attack; BLO, block; CUT, cut; SCA, simultaneous counterattack; ACA, anticipatory counterattack; PCA, posterior counterattack.*

**TABLE 3:** Number and order of occurrences for each pattern in relation to gender.

**Pattern *n*°**	**Lag 0 (FB)**	**Lag 1 (CB)**	**Male (*n*)**	**Order in male**	**Female (*n*)**	**Order in female**
**Focal behavior: offensive actions (attacks) performed by taekwondo athlete A**						
I	OPE	DOD	898	1°	1,164	2°
II	OPE	PCA	231	7°	189	5°
III	DIA	BLO	169	9°	–	–
IV	DIA	CUT	368	4°	477	4°
V	DIA	ACA	112	11°	87	8°
VI	DIA	SCA	831	2°	1,346	1°
VII	DIA	PCA	277	6°	187	6°
VIII	INA	CUT	179	8°	–	–
IX	INA	ACA	132	10°	73	9°
X	INA	SCA	664	3°	788	3°
XI	INA	PCA	298	5°	126	7°
**Focal behavior: offensive actions (counterattacks) performed by taekwondo athlete B**						
XII	ACA	BLO	–	–	23	11°
XIII	ACA	OPE	49	11°	33	10°
XIV	ACA	DIA	94	8°	61	8°
XV	ACA	INA	77	10°	39	9°
XVI	SCA	BLO	114	7°	402	3°
XVII	SCA	OPE	360	3°	410	2°
XVIII	SCA	DIA	562	1°	776	1°
XIX	SCA	INA	412	2°	390	4°
XX	PCA	BLO	78	9°	76	7°
XXI	PCA	DOD	186	6°	237	5°
XXII	PCA	DIA	293	4°	162	6°
XXIII	PCA	INA	191	5°	–	–
**Focal behavior: defensive actions performed by taekwondo athlete B**						
XXIV	BLO	OPE	95	9°	171	6°
XXV	BLO	DIA	156	7°	306	4°
XXVI	BLO	INA	138	8°	121	8°
XXVII	BLO	PCA	66	10°	–	–
XXVIII	DOD	OPE	513	3°	738	2°
XXIX	DOD	DIA	688	1°	1,013	1°
XXX	DOD	INA	555	2°	508	3°
XXXI	CUT	OPE	170	6°	167	7°
XXXII	CUT	DIA	272	4°	280	5°
XXXIII	CUT	INA	177	5°	87	9°
XXXIV	CUT	PCA	–	–	83	10°
Total sequences occurrence			9,405		10,520	

**n*, occurrence; FB, focal behavior; CB, conditioned behavior; OPE, opening; DOD, dodge; DIA, direct attack; INA, indirect attack; BLO, block; CUT, cut; SCA, simultaneous counterattack; ACA, anticipatory counterattack; PCA, posterior counterattack.*

## Discussion

The aim of the present study was to examine the behavioral tactical patterns of Olympic taekwondo competitors, in a sample of male and female competitors, based on Markov processes analysis. The importance of Markov modeling in sports is based on the relevance of decisions made during competitions which require knowledge of the different tactical options, their probability of success, and the risk involved (O’Donoghue, 2010) in choosing the most appropriate option to win the bout. It is well-known that choice reaction time increases as the number of stimulus–response options increases (Borysiuk and Waskiewicz, 2008; Franchini et al., 2008; Kwok, 2012). Such task-specific knowledge structures (stimulus–responses) are acquired through experience (Williams and Elliott, 1999), and are usually referred to as “if-then” statements (McPherson and Thomas, 1989; McPherson, 1994; Gréhaigne and Godbout, 1995; Gréhaigne et al., 2001). They are responsible for the initiation of an appropriate response (“then”) under specified conditions (“if”) (Borysiuk and Waskiewicz, 2008). Markov modeling allows researchers and coaches to know not only specific responses for attacking, counterattacking, and defense actions, but also exhibit a high variety of answers around the entire tactical map described.

To date, this is the first study that analyzed the behavioral tactical pattern of Olympic taekwondo athletes based on the Markov processes analysis for evaluating a competitor’s play in a taekwondo competition. The results showed 32 patterns for male competitors (that explain 9,405 sequences) and 30 patterns for female competitors (that explain 10,520 sequences). Male competitors exhibited 11 sequences initiated by an attack, 11 initiated by a counterattack, and 10 initiated by a defense. Female competitors exhibited 9 sequences initiated by an attack, 11 initiated by a counterattack, and 10 initiated by a defense (Table 3).

In general, the two most frequently used patterns (based on their *z*-values and frequencies of occurrence and expressed as a focal behavior followed by a conditioned behavior) were an opening followed by a dodge (Pattern I) and a direct attack followed by a simultaneous counterattack (Pattern VI). Taekwondo is a sport that has undergone many regulatory changes over the years (Moenig, 2015), and, as a consequence of this dynamism, and the inclusion of electronic chest protectors, appears the opening action (OPE), which has been one of most used tactical action in competition. The results also showed a high variety of patterns, but at the same time, a very specific and well-structured way of competing. That is, the five most frequently used patterns (I, VI, X, XVIII, and XXIX) are similar for both male and female competitors, except that the frequencies of the first and second patterns were exchanged in relation to sex.

The structure of an opening followed by a dodge (Pattern I) could be understood as a pattern intended to test the opponent. That is, it leads attackers to explore the responses of their opponent when the attack is initiated (e.g., giving or cutting distance, or performing a counterattack) (Franchini et al., 2008) and from the point of view of the opponent to see the characteristics of the attack (i.e., sort or long). Meanwhile, the structure of a direct attack followed by a simultaneous counterattack (Pattern VI) can be seen as an option used to stop the opponent’s advance and break the rhythm of combat (Fargas, 1990), so as to obtain a point. That is, when both athletes perform an action with the front leg almost at the same time and both legs crash, the attacker loses balance. As a consequence, the attack of the opponent is stopped. In this sense, athletes require good balance not only to prevent falls and to prevent a warning from the referee (Kyong-go), but also to avoid the dangerous situation of being re-attacked by his or her opponent (Suppiah et al., 2017). In line with some of the previous research (Williams and Elliott, 1999), the first two most frequently used patterns can be understood as actions intended to help recognize, recall, and semantically classify information (as experts with superior skill are able to do) and subsequently to use knowledge of situational probabilities (i.e., expectations) to anticipate future actions (i.e., counterattacks and defensive actions) and facilitate decision-making in terms of speed and accuracy.

In combat sports, when an athlete performs an attack (level 1), the opponent can answer by defending (Patterns I, III, IV, and VIII) or counterattacking (Patterns II, V, VI, VII, IX, X, and XI) (Ottoboni et al., 2014) which corresponds to the level 2 in our tactical scheme. These tactical responses have been classified as defensive or offensive combat styles, respectively (Downey, 2007). A defensive style implies that the opponent opts for facing the combat at a large distance without taking risks (i.e., dodges) and employs defensive actions with the objective of maintaining the score (i.e., cuts and blocks). An offensive style implies that the opponent moves forward, invading the space of the attacker, and competing over a shorter distance by responding to attacks with counterattacks. Both styles are covered by the possible options of significant patterns found in the present study when considering attacks as focal behavior.

Regarding counterattacks as focal behavior, the two most frequently used patterns were the simultaneous counterattack followed (for both sexes) by a direct attack (Pattern XVIII) and the simultaneous counterattack followed in male competitors by an indirect attack (Pattern XIX), and in female competitors by an opening (Pattern XVII). In general terms, all the patterns initiated by a counterattack (level 2) were followed by an attack (level 3), showing the non-stop nature of the bout. It should be noted that not all types of attacks were used by individual athletes in a single bout, as the athletes had their own preferences with respect to the attacks they used (Kwok, 2012). However, it is clear that there is a benefit to a diversity of attack strategies; attackers gain an advantage in that defenders require more time to respond (Menescardi et al., 2015) and therefore experience a decrease in their chances of winning (Suppiah et al., 2017). With these results in mind, taekwondo coaches should consider the benefits of incorporating a high diversity of tactical actions in their training so that the athletes are able to initiate and respond to any tactical option according to the opponent’s characteristics.

With regard to the type of counterattack performed, the most frequently used counterattack was the simultaneous counterattack (Patterns XVI, XVII, XVIII, and XIX). This result is in line with the findings of Menescardi and Estevan (2017) in elite athletes, and in contrast to Falco et al. (2014) and Menescardi et al. (2015) who reported the posterior counterattack as the most frequently used in college athletes. This could be the result of the different sample populations and different regulations over the years. With the introduction of the electronic chest protectors, very minimal contact between the attacker’s foot and the opponent’s protector implied a score (Del Vecchio et al., 2011). A posterior counterattack (Patterns XX, XXI, XXII, and XXIII) implies a lateral or backward movement to avoid the attacker’s technique before kicking him or her, and as a consequence of this movement, the body and guard position of the athlete is changed. Unlike the simultaneous counterattack, the posterior counterattack unbalances the opponent and places them in an unfavorable position from which to kick. This makes them more vulnerable to re-attack and increases the possibility that they will be warned by the referee if they fall to the floor as the result of an attack. Finally, the infrequent use of anticipatory counterattacks (Patterns XII, XIII, XIV, and XV) could be the result of the time necessary to adjust to the opponent’s actions. Anticipatory counterattacks may also increase the risk of counter-counterattacks (Fargas, 1990). Therefore, a more complex calculus has to be performed during the stimulus identification and response selection stages of the information processing model (Kwok, 2012). It is also interesting to note that each type of counterattack was followed by a block (Patterns XII, XVI, and XX) as the most frequently used defense, although the anticipatory counterattack followed by a block was not significant in male competitors (Pattern XII). This highlights the dynamism of the sport and the need to continue with the game after the performance of a tactical action. The use of a block (level 3) after a counterattack (level 2) is a consequence of the attacker’s defensive movement after a kicking action. A block is an action that requires less time to perform than a kicking action; the sequence following a counterattack underlines the fact that it is the attacker who performs this action. Therefore, it is suggested that the attacker practices not only the attack in isolation but also both actions in sequence (an attack with a block) during training. On the other hand, it is suggested that the opponent should feint an attack and hit the side of his or her attacker’s target area by counterattacking him or her, which is an opportunity to score (Borysiuk and Waskiewicz, 2008).

Continuing with defensive actions as a focal behavior, the two most frequently used patterns (Table 3) were a dodge followed by a direct attack, observed in both sexes (Pattern XXIX) and a dodge followed by an indirect attack, in male competitors (Pattern XXX), and by an opening, in female competitors (Pattern XXVIII). These results are in line with González et al. (2011) who observed a frequent use of dodges in competition due to their safety. However, a dodge should be used carefully because athletes can be warned if they move outside of the delineated competition area or if the referee interprets this action as the athlete refusing the match (World Taekwondo Federation [WTF], 2012). This can be observed in the frequency of occurrence of Patterns I, IV, VIII, XII, XVI, and XX. The frequency of use of Patterns XII, XVI, and XX highlights the fact that a block was more often used after a counterattack, while a dodge or a cut was more often used after an attack (Patterns I, IV, and VIII). These results are in line with Fargas (1990), who stated that blocks are employed to avoid being kicked when no other options are possible in close combat. The rest of the observed patterns suggested that during interchanges of kicks and punches, defensive actions (such as dodges, cuts, or blocks) were also used to avoid being kicked and scored upon, which accords with the internal logic of this sport (Fargas, 1990). In short, it is suggested that when the attacker initiates the sequence he or she observes the movement of the opponent, because the opponent has two options: the opponent can either move forward (and decrease the distance) or backward (and increase the distance with a dodge). In both cases, the sequence is stopped and the rhythm of combat is broken, and a new attack is needed to initiate it. If the attacker does not observe either of these two actions, the attacker must be ready to block the opponent’s counterattack.

In summary, the results of the present study showed a total of 62 significant patterns. This demonstrates that elite athletes need to master a huge diversity of tactical possibilities, including not only attacks and counterattacks but also defensive actions. In this sense, and in line with Downey (2007), it seems that the best athletes are more technically sophisticated than instinctually savage and that fight-ending techniques are sometimes quite subtle. It would be interesting to know which are the most effective and this should be the focus of future studies. Lastly, there were six patterns that were not significant to one sex; future studies should focus on understanding why they are not used. One of the reasons could be the fact that the study has analyzed the patterns of elite athletes. That is, experts, compared to novices, focus on higher-level concepts, have made more connections between these concepts, and seem to be more capable of using the more appropriate action considering the game’s goal structure. Therefore, it would be interesting to compare the sample with other levels of athletes to understand whether different level athletes have different patterns.

It is suggested that coaches should plan and introduce into the training sessions all the observed sequences, with a special focus on the tactical actions that are most frequently performed during competition and the ability to recognize and respond to them properly. That is, among male competitors, 11 sequences were initiated by an attack, 11 were initiated by a counterattack, and 10 sequences were initiated by a defense. The five most popular sequences were as follows: (a) an opening with a dodge, (b) a direct attack and simultaneous counterattack, (c) a dodge with a direct attack, (d) an indirect attack with a simultaneous counterattack, and (e) a simultaneous counterattack with a direct attack. Meanwhile, among female competitors, nine sequences were initiated by an attack, 11 were initiated by a counterattack, and the 10 initiated by a defense. The five most popular sequences were as follows: (a) a direct attack and simultaneous counterattack, (b) an opening and dodge, (c) a dodge and direct attack, (d) an indirect attack and simultaneous counter attack, and (e) a simultaneous counterattack and direct attack. Moreover, coaches should focus on the continuity of the sequence by counterattacking and blocking the attacker’s action, in addition to focusing on ways to break the rhythm of combat (i.e., by cutting or blocking), as it has been explained throughout the manuscript. Lastly, coaches should take into account the patterns that are similar between both sexes and those that are different when planning training, in order to tailor the training to the athlete’s sex and the session goal (attacks, counterattacks, or defensive actions). That is, training sessions should have a double goal, both to train common patterns and to train male and female competitors separately according to the different patterns of each sex.

## Conclusion

In the current study, a Markov chain approach was applied to study taekwondo interactions between competitors, which goes beyond earlier studies on this sport. Markov chains provide coaches and researchers with relevant information not only about the frequency of the occurrence of certain actions, as in the majority of research on taekwondo, but also provides information about the order in which those actions were performed during real competition. In the current study, there were 32 significant sequences among male competitors and 30 among female competitors. The most used sequences for both sexes were an opening followed by a dodge and attack (direct or indirect) followed by simultaneous counterattack. The results show that the dodge was the most frequently deployed defensive action against an attack while the simultaneous counterattack was the most frequently used counterattack. Therefore, coaches and athletes should focus on these patterns during training to prepare for the demands of real competition.

## Data Availability Statement

The datasets generated for this study are available on request to the corresponding author.

## Author Contributions

CM participated in the study design and data collection, conducted the statistical analyses and contributed to the interpretation of the results, drafted the manuscript, and approved the final manuscript as submitted. CM, CF, and AH-M conceived the study; participated in its design and coordination; contributed to the video coding, the data collection, and the interpretation of results; drafted the manuscript; and approved the final manuscript as submitted. CR and VM-S participated in the study design, contributed to the interpretation of the results, reviewed and provided feedback to the manuscript, and approved the final manuscript as submitted. All authors made substantial contributions to the final manuscript.

## Conflict of Interest

The authors declare that the research was conducted in the absence of any commercial or financial relationships that could be construed as a potential conflict of interest.
